# Recognition Patterns of the C1/C2 Epitopes Involved in Fc-Mediated Response in HIV-1 Natural Infection and the RV114 Vaccine Trial

**DOI:** 10.1128/mBio.00208-20

**Published:** 2020-06-30

**Authors:** William D. Tolbert, Verna Van, Rebekah Sherburn, Marina Tuyishime, Fang Yan, Dung N. Nguyen, Sherry Stanfield-Oakley, David Easterhoff, Mattia Bonsignori, Barton F. Haynes, M. Anthony Moody, Krishanu Ray, Guido Ferrari, George K. Lewis, Marzena Pazgier

**Affiliations:** a Infectious Disease Division, Department of Medicine of Uniformed Services University of the Health Sciences, Bethesda, Maryland, USA; b Division of Vaccine Research of Institute of Human Virology, University of Maryland School of Medicine, Baltimore, Maryland, USA; c Department of Surgery, Duke University Medical Center, Durham, North Carolina, USA; d Duke Human Vaccine Institute, Duke University School of Medicine, Durham, North Carolina, USA; e Department of Medicine, Duke University School of Medicine, Durham, North Carolina, USA; f Department of Pediatrics, Duke University School of Medicine, Durham, North Carolina, USA; g Department of Molecular Genetics and Microbiology, Duke University Medical Center, Durham, North Carolina, USA; McMaster University

**Keywords:** RV144, human immunodeficiency virus, structural biology, vaccines

## Abstract

Antibodies (Abs) specific for CD4-induced envelope (Env) epitopes within constant region 1 and 2 (C1/C2) were induced in the RV144 vaccine trial, where antibody-dependent cellular cytotoxicity (ADCC) correlated with reduced risk of HIV-1 infection. We combined X-ray crystallography and fluorescence resonance energy transfer-fluorescence correlation spectroscopy to describe the molecular basis for epitopes of seven RV144 Abs and compared them to A32 and C11, C1/C2 Abs induced in HIV infection. Our data indicate that most vaccine Abs recognize the 7-stranded β-sandwich of gp120, a unique hybrid epitope bridging A32 and C11 binding sites. Although primarily directed at the 7-stranded β-sandwich, some accommodate the gp120 N terminus in C11-bound 8-stranded conformation and therefore recognize a broader range of CD4-triggered Env conformations. Our data also suggest that Abs of RV144 and RV305, the RV144 follow-up study, although likely initially induced by the ALVAC-HIV prime encoding full-length gp120, matured through boosting with truncated AIDSVAX gp120 variants.

## Abstract (precis)

Antibody-dependent cellular cytotoxicity (ADCC) correlated with a reduced risk of infection from HIV-1 in the RV144 vaccine trial, the only HIV-1 vaccine trial to date to show any efficacy. Antibodies specific for CD4-induced envelope (Env) epitopes within constant region 1 and 2 (cluster A region) were induced in the RV144 trial and their ADCC activities were implicated in the vaccine efficacy. We present structural analyses of the antigen epitope targets of several RV144 antibodies specific for this region and C11, an antibody induced in natural infection, to show what the differences are in epitope specificities, mechanism of antigen recognition, and ADCC activities of antibodies induced by vaccination and during the course of HIV infection. Our data suggest that the truncated AIDSVAX gp120 variants used in the boost of the RV144 regimen may have shaped the vaccine response to this region, which could also have contributed to vaccine efficacy.

## INTRODUCTION

Human immunodeficiency virus 1 (HIV-1) is unique as a virus, as it combines two characteristics making it almost always fatal if untreated and, with two exceptions, incurable: a high mutation frequency and the ability to integrate into cellular DNA and form a latent reservoir. This leaves the immune system with only two options: block infection completely or clear all infected cells. One of the main mechanisms by which the immune system could potentially accomplish the latter is by antibody-dependent cellular cytotoxicity (ADCC), where antibodies recognizing the virus serve as the bridge to the immune system for the killing of infected cells. The HIV-1 envelope glycoprotein trimer (Env), a gp41-gp120 heterodimer, is the only universally exposed viral protein on the surface of HIV-1 and, therefore, the lone target available for recognition by the immune system. Env changes conformation to facilitate viral entry upon binding to CD4 on target T cells. This leads to the exposure of highly conserved CD4-inducible (CD4i) epitopes within constant region 1 and 2 (C1/C2) of gp120, known as the cluster A region, which contains non-neutralizing (as assessed using assays measuring direct neutralization) but potent ADCC epitope targets for antibodies (1–5). The cluster A epitopes become exposed upon Env triggering with cell surface CD4 and, therefore, are available for antibody recognition during the viral entry process in active infection of the CD4^+^ T cell population and in cell-to-cell spread (1, 6–9). It is well documented that anti-cluster A antibodies are induced over the course of natural infection and contribute a significant portion of the overall ADCC activity from the sera of infected individuals (1, 4, 10–13). In recent years, several monoclonal antibodies (mAbs) specific for the cluster A region have been isolated from subjects with natural infection, allowing for the structural analysis and fine epitope mapping of this region. The prototype antibodies for this region include A32 and C11 (14, 15), mAbs that recognize distinct non-overlapping epitopes (15, 16). In recent years, we have reviewed the structure and function of A32 and other antibodies isolated from HIV-1-infected subjects that overlap A32 and C11 in binding to Env (2–5). In contrast to A32, where the epitope structure was determined by crystallography of its antigen binding fragment (Fab)-antigen complex (3), the C11 epitope was mapped indirectly by structural analysis of the C11-like antibody N12-i3 (5). This structure revealed a unique property of N12-i3, and by inference, C11, in that they bind to the gp120 N terminus in a previously unseen 8-stranded β-sandwich conformation. Interestingly, our and others’ functional studies uniformly indicate that antibodies recognizing the cluster A region are able to induce cytolysis of gp120-coated target cells (1, 2, 9, 10) and HIV-infected cells which retain CD4 at their surface or are triggered by CD4 mimetics (17–21), confirming their potential for therapeutic or preventative treatment of HIV-1 infection.

The cluster A region was also recently implicated in the reduced risk of infection in the ALVAC-HIV/AIDSVAX-B/E RV144 vaccine trial, the only trial to date that resulted in significant vaccine efficacy (31.2%) (22) and that demonstrated, for the first time, that vaccination can protect humans from HIV-1 infection. Protection in the RV144 vaccine trial was due to the generation of gp120-specific antibodies with limited neutralizing activity in the presence of very modest cytotoxic T cell responses (22, 23). Interestingly, in vaccine recipients with low levels of IgA, ADCC—together with other variables—correlated with a reduced risk of infection, suggesting that ADCC may have contributed to protection (23). Epitope mapping of antibodies induced in the trial indicated a narrow specificity with most of the ADCC responses targeting epitopes within the V2 loop (24) and the cluster A (25) regions of gp120. Nineteen of 20 CD4i mAbs, isolated from memory B cells of 6 vaccinated individuals, targeted conformational, A32-blockable epitopes in the cluster A region and mediated ADCC but did not directly neutralize the virus in a pseudovirus-TZM-bl assay (25). Subsequent studies demonstrated that vaccine-induced plasma IgA and cluster A mAbs of the IgA isotype from vaccine recipients blocked ADCC effector functions on infected CD4^+^ T cell targets by HIV-1 Env IgG (26). Cluster A-specific antibodies synergized with vaccine-induced V2 loop ADCC-mediating antibodies in the killing of target cells by ADCC (27). Interestingly, these antibodies were also capable of ADCC activity against clades that were not represented in the vaccine immunogen. Furthermore, results from the recent RV144 follow up study, referred to as RV305 (ClinicalTrials.gov registration no. NCT01435135), in which a subset of RV144 vaccinees were immunized again with the same RV144 vaccine components, indicated that A32-like ADCC responses induced by ALVAC/AIDSVAX B/E vaccine could be effectively boosted. Sera from RV305 subjects were capable of effective ADCC, and several isolated A32-like mAbs showed increased potency and breadth compared to those of A32 and mAbs isolated from RV144 subjects (28).

Specificities of RV144 antibodies to the cluster A region were assessed based on competition for binding to the Env antigen with A32 Fab (25), while competition with C11 was not tested. To better understand the epitope specificity of the anti-cluster A response induced in this trial, we determined the Env antigen-Fab complex crystal structures of two cluster A RV144 antibodies, CH54 and CH55. In addition, to analyze how the specificity of the vaccine-induced anti-cluster A response compared to that of the anti-cluster A response induced in natural infection, we solved the crystal structure of the gp120 antigen complex of C11, therefore defining the recognition site of the second prototype antibody of the cluster A region. Finally, we employed a combined fluorescence resonance energy transfer-fluorescence correlation spectroscopy (FRET-FCS) approach (3) to generate data that, combined with information gained from structural studies, allowed us to map the Env recognition sites of 5 additional cluster A antibodies isolated from RV144 vaccinees. Altogether, our results define differences in the Env sites/conformations targeted by cluster A antibodies induced in natural infection compared to those induced by vaccine regimens in the RV144 and RV305 vaccine trials. The cluster A epitopes from natural infection and vaccination only partially overlap the vaccine-induced response largely biased toward epitopes within the 7-stranded β-sandwich of the gp120 inner domain.

## RESULTS

### ADCC activities of cluster A antibodies induced in natural infection and in the RV144 ALVAC/AIDSVAX B/E vaccine regimen.

**FIG 1:**
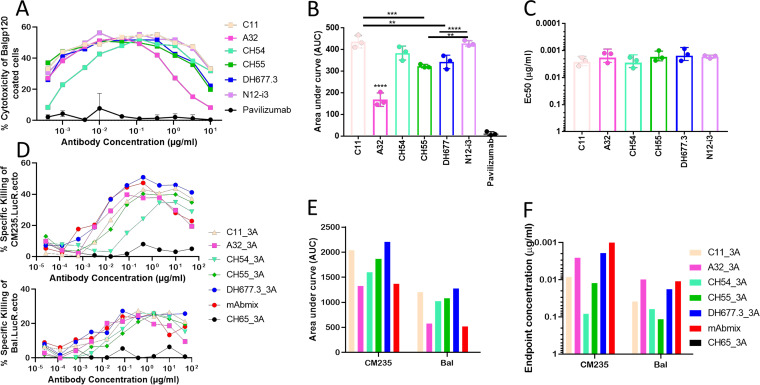
ADCC activity of vaccine-induced and naturally occurring HIV-1 antibodies. (A) Cytotoxicity curve of GFP-CEM-NKR-CCR5-SNAP cells coated with BaL gp120. CH54, CH55, DH677.3, N12-i3, A32, C11, and negative-control palivizumab were tested for ADCC potency over a broad range of concentrations (10 μg/ml to 0.0004 μg/ml) using human PBMCs as effector cells. Cytotoxicity was defined as effector cells which were GFP-CCR5^+^. *N* = 3, means ± standard errors of the means (SEMs). (B) Areas under the curve (AUCs) for all tested antibodies. (C) The concentration of antibody at which half maximal cytotoxicity occurs; EC_50_ (μg/ml). Each antibody was assayed in triplicates, all data were analyzed using GraphPad Prism version 7.05. **, *P* < 0.005; ***, *P* < 0.001; ****, *P* < 0.0001 as determined by one-way analysis of variance (ANOVA) with *post hoc* Tukey test. (D) The percent specific killing of CEM-NKR cells infected with HIV-1 CM235 (top) or BaL (bottom) as targets (T) and PBMCs from an HIV-1-seronegative individual as effectors (E) with E:T ratio of 30:1. C11, A32, CH54, CH55, DH677.3, mAbmix, and negative-control CH65 were tested with 50 μg/ml starting concentration and subsequent 1:5 dilutions. The titration curves represent the killing 6 h post-incubation of T+E+mAbs. (E) Areas under the curves (AUCs) for all tested antibodies. (F) Endpoint concentrations (μg/ml), the concentration at which at least 15% target cell killing occurs. The data represent a single experiment with each mAb dilution tested in duplicates. CH65 antibody and mAb mix (A32, 2G12, CH44, and 4E10) used as negative and positive controls, respectively.

Both the CH54 and CH55 antibodies, isolated from an RV144 ALVAC/AIDSVAX B/E vaccine recipient, and DH677.3, isolated from an RV305 vaccine recipient, have been well characterized in terms of ADCC breadth and potency; however, these were conducted in separate experiments. To allow a direct comparison between antibodies induced by vaccine regimens and natural infection, we tested CH54, CH55, DH677.3, N12-i3, and the two prototype antibodies of cluster A, A32 and C11, in two well-documented ADCC assays. Using the modified rapid fluorometric ADCC (RFADCC) assay targeting green fluorescent protein (GFP)-CEM-NKR-CCR5-SNAP cells (29) coated with Bal gp120, derived from a clade not present in the vaccination regimen, both antibodies from natural infection and from vaccination showed similar maximum lysis ranging from 52.54% for CH55 to 58.4% for N12-i3 (Fig. 1A). CH54 was active over the narrowest range of tested concentrations; however, the area under the curve (AUC) was significantly higher than that of the prototype antibody A32 (383.7 versus 155.8, respectively, *P* < 0.0001) and did not significantly differ from those of other tested vaccine-induced (CH55, 318.2; DH677.3, 347.7) or naturally occurring (C11, 436; N12-i3, 418.4) antibodies. Overall, all antibodies assayed in this clade-mismatched entry target model of ADCC mediated significant cell lysis with similar potency, as demonstrated by the comparable 50% effective concentration (EC_50_) values (Fig. 1A to C).

To further test the potency of antibodies elicited by vaccination versus those from natural infection, we tested the panel for ADCC activity using HIV-infected CEM-NKR-CCR5 cells as targets in a luciferase-based assay (27). Peripheral blood mononuclear cells (PBMCs) isolated from an HIV-1-seronegative individual were used as effector cells and tested against subtype B HIV-1_BaL_- or HIV-1_CM235_-infected target cells in the presence of serial dilutions of mAbs. The mAbs of interest mediated ADCC against cells infected with both Nef-deficient HIV-1 CM235 and BaL infectious molecular clone (IMC) (Fig. 1D). Overall, we observed higher AUC against the HIV-1 CM235 than the BaL-infected cells for each mAb. Vaccine-induced CH54, CH55, and DH677.3 mAb AUCs were higher than the A32 mAb AUC against both sets of infected target cells, whereas only the DH677.3 AUCs were higher than both the A32 and C11 mAb AUCs (Fig. 1E). The analysis of the endpoint concentrations, the lowest antibody concentration at which specific killing was at least 15%, revealed a different profile for the A32 mAb which mediated ADCC at concentrations lower than that of any of the vaccine-induced mAbs against both infected cell targets. C11 mAb endpoint concentrations (ECs) were higher than CH54 and CH55 mAb ECs but lower than DH677.3 ECs against both infected target cell types (Fig. 1F). The profiles of ADCC against infected cells are more heterogeneous than those observed with the gp120-coated target cells. As discussed below, this is most likely the result of a higher polymorphism of envelope conformational structures expressed on the surfaces of infected cells.

### Crystal structure of C11 Fab in complex with gp120_93TH057_ (S31C, N80C) core_e_+N/C.

The cluster A epitope region is defined as a combined recognition site of two prototype antibodies isolated from natural infection, mAb A32 and C11. Of the two, only the A32 epitope region had been defined at atomic level (4); the epitope structure of C11 has remained unsolved. We recently reported the structure of the C11-like antibody Fab, N12-i3, in complex with gp120 antigen (5), which indirectly defined the C11 epitope region. Crystallization of the prototypic C11 antibody Fab in complex with the gp120 antigen had eluded us despite numerous attempts with various gp120 antigens and Fab combinations. mAb N12-i3 was derived from memory B cells isolated from a natural viral suppressor (NVS), an HIV-1-infected individual who suppressed HIV-1 replication to <400 copies/ml in the absence of antiretroviral therapy (5, 30–32), and competed for C11, but not A32, in binding to Env in enzyme-linked immunosorbent assay (ELISA) competition experiments (1). Our structural analyses indicated that N12-i3 binds to a unique gp120 conformation in which the N terminus forms an 8th strand to the 7-stranded β-sandwich of the gp120 inner domain (5). The gp120 N terminus can also form a short 2-stranded β-sheet with the C terminus (16) that is more similar to its conformation when bound to gp41 in the Env trimer (33–35). To lock the N terminus in the conformation observed in the N12-i3 complex structure and, therefore, presumably the C11 conformation, we added a stabilizing disulfide bond between gp120 residues 31 and 80 (S31C and N80C). We were able to express this mutant clade A/E 93TH057 gp120 extended core (core_e_) with N and C termini (gp120_93TH057_ [S31C, N80C] core_e_+N/C) at similar levels to those of the unmodified protein. Binding analyses using surface plasmon resonance (SPR) confirmed improved binding of C11 to the gp120 core_e_+N/C mutant than to the wild type with a dramatic decrease in the *k*_on_ association constant, a decrease in the *k*_off_, and an overall slight increase in the affinity constant, *K_D_* (see Table S1 in the supplemental material). According to the same protocol for complex formation, we were able to generate a C11 Fab-gp120_93TH057_ (S31C, N80C) core_e_+N/C complex and grow crystals to diffraction quality. These crystals diffracted to 3.45 Å and belonged to space group P2_1_2_1_2_1_ with cell dimensions a = 89.4 Å, b = 111.0 Å, and c = 217.6 Å, with two C11 Fab-gp120_93TH057_ (S31C, N80C) core_e_+N/C complexes in the asymmetric unit. Surprisingly, we were not able to find the CD4 mimetic M48U1 in the structure, suggesting that the crystal lattice contacted and excluded M48U1 from binding. In support of this interpretation, we were able to reproduce crystals in the absence of M48U1 more easily than in its presence. In addition, we also solved the 2.7-Å resolution crystal structure of C11 Fab alone (see Fig. S1 and S2). Combined data collection and refinement statistics are shown in Table 1.

**TABLE 1:** Data collection and refinement statistics

Parameter	Value				
	C11 Fab-gp120_93TH057_ (S31C, N80C) core_e_+N/C	CH54 Fab-gp120_93TH057_ core_e_+N/C-M48U1	CH55 Fab-gp120_93TH057_ core_e_	C11 Fab	CH55 Fab
Data collection^*a*^					
Wavelength (Å)	0.979	0.979	0.979	1.033	0.979
Space group cell parameters	P212121	C2	P6_1_22	C2	P1
a, b, c (Å)	89.4, 111.0, 217.6	174.8, 42.2, 119.8	90.6, 90.6, 409.0	134.5, 56.0, 70.7	74.0, 74.0, 75.9
α, β, γ (°)	90, 90, 90	90, 115.2, 90	90, 90, 120	90, 106.9, 90	106.8, 108.4, 91.8
Complexes (AU)	2	1	1	1	3
Resolution (Å)	50–3.45 (3.51–3.45)	50–2.9 (2.95–2.9)	50–3.8 (3.92–3.8)	50–2.7 (2.75–2.7)	70–2.46 (2.59–2.46)
No. of reflections					
Total	71,966	54,282	29,485	75,724	73,117
Unique	26,654	16,963	8,672	14,023	40,400
R_merge_^*b*^ (%)	16.2 (68.5)	17.4 (83.3)	24.9 (92.5)	14.6 (75.2)	8.5 (69.7)
R_pim_^*c*^ (%)	11.7 (49.6)	11.6 (54.4)	14.7 (56.7)		8.5 (69.7)
*CC_1/2_*^*d*^	0.98 (0.55)	0.98 (0.48)	0.98 (0.45)		
I/σ	6.6 (1.1)	11.1 (1.0)	4.7 (1.0)	20.9 (1.3)	4.9 (1.2)
Completeness (%)	91.3 (89.6)	95.9 (97.3)	81.9 (89.6)	97.3 (87.3)	76.8 (79.4)
Redundancy	2.7 (2.5)	3.2 (3.1)	3.4 (3.3)	5.4 (4.7)	1.8 (1.8)
Refinement statistics					
Resolution (Å)	50.0–3.45	50.0–2.9	50.0–3.8	50–2.7	42–2.46
R^*e*^ (%)	23.1	25.0	27.1	25.3	25.9
R_free_^*f*^ (%)	28.4	30.7	32.6	31.3	31.4
No. of atoms					
Protein	11,734	5,995	5,717	3,323	9,756
Water				10	84
Ligand/ion	406	172	140	15	
Overall B value (Å)^2^					
Protein	113	90	111	96	68
Water				206	50
Ligand/ion	162	101	128	231	
RMSD^*g*^					
Bond lengths (Å)	0.004	0.005	0.005	0.009	0.005
Bond angles (°)	0.8	1.5	1.1	1.4	0.9
Ramachandran^*h*^ (%)					
Favored	82.5	84.0	86.8	80.7	87.6
Allowed	14.1	11.3	10.4	13.0	9.5
Outliers	3.4	4.7	2.8	6.3	2.9
PDB ID	6OEJ	6MG7	6OFI	4FZ8	6OED

a Values in parentheses are for highest-resolution shell. AU, arbitrary units.

b *R*_merge_ = ∑│*I* − <*I*>│/∑*I*, where *I* is the observed intensity and <*I*> is the average intensity obtained from multiple observations of symmetry-related reflections after rejections.

c R_pim_ as defined by Weiss (65).

d *CC_1/2_ *as defined by Karplus and Diederichs (66).

e *R* = ∑║Fo│-│ Fc║/∑│Fo│, where Fo and Fc are the observed and calculated structure factors, respectively.

f R_free_ as defined by Brünger (67).

g RMSD, root mean square deviation.

h Calculated with MolProbity.

10.1128/mBio.00208-20.1 FIG S1 Crystal structure of C11 Fab-gp120_93TH057_ (S31C, N80C) core_e_+N/C gp120 complex and CH55 Fab. (A) Structural comparison of crystallographic copies of C11 Fab-gp120_93TH057_ (S31C, N80C) core_e_+N/C complex present in asymmetric unit of crystal. gp120 is colored with a layered architecture, and the C11 CDRs are colored as labeled. The root mean squared distance (RMSD) between the two complex copies is 0.824 Å for main chain atoms. The C11 Fab is glycosylated on heavy chain asparagine 52A (Kabat numbering), which is largely disordered in the structure. While the glycan that is visible in the structure makes no contact to gp120, a similarly placed wild-type glycan could pack against the gp120 C terminus and potentially aid in binding. (B) Structural comparison of crystallographic copies of CH55 Fab present in asymmetric unit of crystal. The RMSD for main chain atoms for the three CH55 Fabs in the asymmetric is 0.925 Å. In all three copies, an identical portion of the CDR H3 is disordered but continuous density is present for all other heavy- and light-chain CDRs. Download FIG S1, PDF file, 0.1 MB. Copyright © 2020 Tolbert et al. 2020 Tolbert et al. This content is distributed under the terms of the Creative Commons Attribution 4.0 International license.

10.1128/mBio.00208-20.2 FIG S2 Structural comparison of C11 and CH55 Fabs from antigen complex and apo crystal structures. (A) Superimposition of the two copies of C11 Fab from the C11 Fab-gp120_93TH057_ (S31C, N80C) core_e_+N/C gp120 complex to the C11 Fab from the apo structure (PDB code 4FZ8). Much of the difference between the bound and unbound C11 Fab originates from the constant part of the Fab (C_H_ and C_L_), which is in a different relative position in the two crystals. The average main chain RMSD for the full fab is 2.92 Å, but for just the variable part, it is only 0.679 Å. The CDRs are ordered in the unbound C11 Fab and largely superimposable with the bound C11 Fab conformations, implying that there are only small conformational changes necessary for binding. (B) Superimposition of CH55 Fab from CH55 Fab-gp120_93TH057_ core_e_ complex to the three copies of CH55 Fab from the apo structure. The constant part of the CH55 Fab accounts for much of the difference between the bound and unbound structures, with an average main chain RMSD of 2.48 Å for the full Fab and 1.06 Å for the variable part. Aside from the CDR H3, which is only ordered in the complex structure, the CDRs are largely superimposable for both the bound and unbound structures. However, CDR H3 undergoes a significant rearrangement upon binding to gp120. (C) The C11, CH54, and CH55 Fab residues involved in gp120 binding (Fab buried surface and contact residues) are shown over the primary sequence of each Fab. Residues buried at the surface interface as determined by PISA (https://www.ebi.ac.uk/msd-srv/prot_int/pistart.html) are shown in grey, and contact residues as defined by a 5-Å distance criterion cutoff are shown immediately above the Fab residue; main chain (−), side chain (+), and both main and side chain (±) interactions are colored based on contact type: hydrophobic, green; hydrophilic, blue; or both, black. CDRs are colored as in panels A and B. Fab residues are numbered with Kabat numbering with insertions as indicated. Download FIG S2, PDF file, 0.2 MB. Copyright © 2020 Tolbert et al. 2020 Tolbert et al. This content is distributed under the terms of the Creative Commons Attribution 4.0 International license.

10.1128/mBio.00208-20.5 TABLE S1 Binding kinetics of mAb C11 to gp120 core_e_+N/C termini and gp120 core_e_+ N/C termini with S31C/N80C mutation measured by SPR. The assay was run by passing Env glycoproteins over the immobilized antibody at 0 to 200 nM concentrations as described in Materials and Methods. The binding kinetics (association rates [*k_a_*], dissociation rates [*k_d_*], and affinity constants [*K_D_*]) were calculated with the BIAevaluation software. Standard deviations of *k_a_*, *k_d_*, and *K_D_* for two experiments are shown. Download Table S1, DOCX file, 0.1 MB. Copyright © 2020 Tolbert et al. 2020 Tolbert et al. This content is distributed under the terms of the Creative Commons Attribution 4.0 International license.

**FIG 2:**
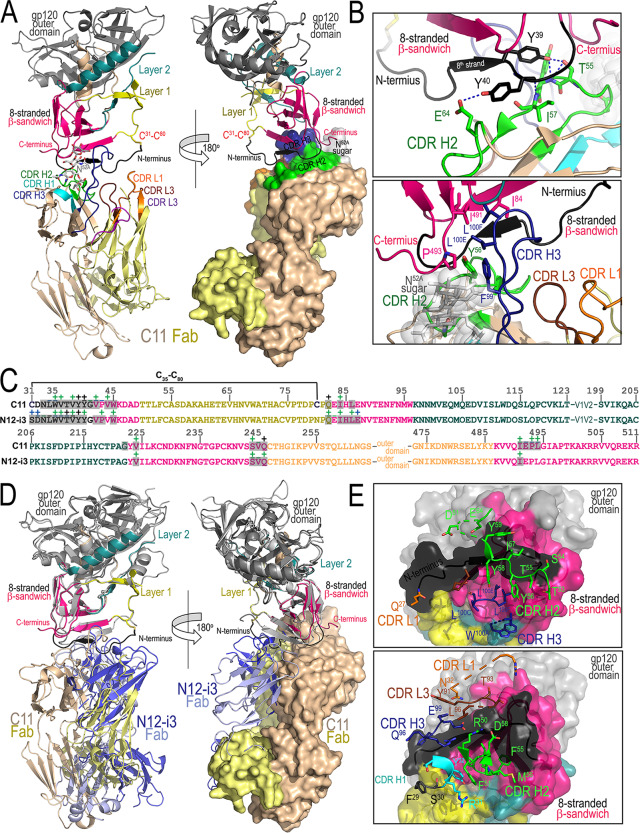
Crystal structure of C11 Fab-gp120_93TH057_ (S31C, N80C) core_e_+N/C gp120 complex. (A) The overall structure of the complex is shown as a ribbon diagram and with molecular surface displayed over Fab molecule (a 180° view). The gp120 inner domain is colored black-pink for 8-stranded β-sandwich, yellow for layer 1, and cyan for layer 2. The N terminus of gp120 (residues 33 to 42) is colored in black. CDRs of C11 Fab are colored as indicated. (B) Key contacts of C11 to the 8-stranded β-sandwich are mediated by its CDR H2 and H3. The sugar at position 52A of CDR H2 of C11 is shown as sticks and spheres. Hydrogen bonds are shown as dotted lines. (C) Epitope footprints of C11 and N12-i3. The gp120 residues involved in Fab binding (gp120 buried surface and contact residues) are shown over the primary sequence of gp120. Residues buried at the surface interfaces as determined by PISA (https://www.ebi.ac.uk/msd-srv/prot_int/pistart.html) are shown in red and contact residues as defined by a 5-Å distance criterion cutoff are shown immediately above the gp120 residue; main chain (−), side chain (+), and both main and side chain (±) interactions are colored based on contact type: hydrophobic, green; hydrophilic, blue; or both, black. (D) Structural comparisons of gp120 antigen complexes of C11 and N12-i3 Fabs. Structures of C11 Fab-gp120_93TH057_ (S31C, N80C) core_e_+N/C gp120 and N12-i3 Fab/N5-i5Fab-gp120_93TH057_ core_e_+N/C (PDB code 5W4L) (5) complexes were superimposed based upon gp120. Only the Fab of N12-i3 is shown for N12-i3 Fab/N5-i5Fab-gp120_93TH057_ core_e_+N/C and its gp120 is colored in gray. (E) C11 Fab and N12-i3-gp120 antigen interface. The molecular surface is displayed over gp120 and is colored as in panel A. C11 Fab (top) and N12-i3 Fab (bottom) residues that contribute to gp120 binding are shown as sticks. Complexes are shown in the same orientation.

C11 binds almost exclusively to the gp120 inner domain β-sandwich, and slightly more than 50% of the gp120 buried surface area (BSA) is made to the N-terminal 8th β-strand of the sandwich (gp120 residues 33 to 42) (Fig. 2A; see also Table S2). This is reminiscent of how N12-i3 binds gp120, with approximately 65% of its gp120 BSA devoted to the 8th β-strand. The gp120 N terminus in the C11-bound conformation folds into a short β-strand (residues 37 to 39) that docks parallel to the 7th stand of the β-sandwich with the remaining portion of the N terminus, assuming a random coil restrained by the introduced C-31 to C-80 bond (Fig. 2B). The C-31 to C-80 bridge cross-links the very N-terminal end of gp120 to the 7-stranded β-sandwich. Interestingly, C11 binds at the top of the β-sandwich in an orientation that also allows engagement of the gp120 C terminus. We resolved densities for gp120 residues 492 to 497 that direct the gp120 C terminus at a 90° angle relative to the N terminus (Fig. 2B). C11 contacts this region primarily through a glycosyl group on the heavy chain (N^52^, Kabat numbering); the contribution of the N52 glycan to binding was confirmed by analyses of deglycosylated Fabs (data not shown). In addition to contacting the 8th β-strand, C11 establishes contacts with other residues of the β-sandwich (residues 44, 84, 86, 224, 244 to 246, 491, and 492), with very few contacts to the gp120 inner domain mobile layer 1 (residue 82) and layer 2 (residues 221 and 222) (Fig. 2C). Interestingly, most of these contacts are split almost equally by 17-residue-long complementarity-determining region (CDR) H2 and 22-residue-long CDR H3 that contribute the majority of BSA to the complex (685 Å^2^ of 811 Å^2^ total buried exclusively by the heavy chain) (Table S2). The light-chain contacts are limited to the gp120 N terminus, as it must accommodate the random coil formed at the very end of the gp120 C terminus.

10.1128/mBio.00208-20.6 TABLE S2 Details of the C11, CH54, CH55, A32, and N12-i3 interfaces based on the C11 Fab-gp120_93TH057_ core_e_+N/C, C54 Fab-gp120_93TH057_ core_e_+N/C-M48U1, CH55 Fab-gp120_93TH057_ core_e_, A32 Fab-ID2_93TH057_, and N12-i3 Fab-gp120_93TH057_ core_e_+N/C-M48U1 structures as calculated by the EBI PISA server (https://www.ebi.ac.uk/msd-srv/prot_int/pistart.html). The two copies in the asymmetric unit of the C11, A32, and N12-i3 complexes are averaged in the table. Download Table S2, DOCX file, 0.1 MB. Copyright © 2020 Tolbert et al. 2020 Tolbert et al. This content is distributed under the terms of the Creative Commons Attribution 4.0 International license.

The large area buried at the N-terminal 8th β-strand explains the dependence of C11 and N12-i3 on a full-length gp120 N terminus for binding (Table S2). The dependence on the gp120 N terminus is not the only similarity between C11 and the C11-like antibody N12-i3: both greatly superimpose in how they approach the antigen, with slight differences in relative orientations of their heavy and light chains (Fig. 2D and E). C11 buries a total of 1,579 Å^2^, while N12-i3 buries 1,656 Å^2^, with much of the difference corresponding to a greater BSA to the N terminus for N12-i3 versus that for C11. In both cases, contacts to gp120 N terminus are made predominantly through CDR H2, with noticeable differences in how the gp120 inner domain β-sandwich is gripped from the side. C11 sits “more at the top” of the β-sandwich, whereas N12-i3 approaches this region from the side. In addition, there are also noticeable differences in the conformation of the N terminus in the 8-stranded β-sandwich between C11- and N12-i3-bound conformations of gp120 (see Fig. S3). In the N12-i3-bound conformation, the 8th strand spans a longer stretch of the N terminus (residues 35 to 39) and docks perfectly parallel to the sandwich to form a flat 8-stranded β-sheet structure; the 8th strand in C11-bound conformation is shorter (residues 37 to 39), with the very N terminus assuming a random coil conformation due to the introduced C-31 to C-80 bond. Finally, C11 only makes minor contacts with inner domain mobile layers 1 and 2 that form the basis for the epitope of A32 and the other A32-like antibodies (2–4).

10.1128/mBio.00208-20.3 FIG S3 Conformation of gp120 core_e_+N/C termini in C11 and N12-i3 bound state. gp120 from complexes C11 Fab-gp120_93TH057_ (S31C, N80C) core_e_+N/C gp120 (left) and N12-i3 Fab-gp120_93TH057_ core_e_+N/C gp120 (right) is shown and colored grey for gp120 outer domain, pink for 7-stranded β-sandwich, yellow for layer 1, and cyan for layer 2. The N terminus of gp120 (residues 33 to 42) is colored in black. Complexes are shown in the same orientation and the C-31 to C-80 disulfide bond is shown as sticks. Download FIG S3, PDF file, 0.1 MB. Copyright © 2020 Tolbert et al. 2020 Tolbert et al. This content is distributed under the terms of the Creative Commons Attribution 4.0 International license.

### Crystal structures of RV144 CH54 Fab and CH55 Fab in complex with clade A/E 93TH057 gp120 antigen.

To map the exact Env recognition sites of the RV144 vaccine-induced mAbs CH54 and CH55 and assess if these differ from the epitopes of cluster A antibodies A32 and C11 induced in the course of natural infection or from those induced in the RV305 trial (28), we determined crystal structures of complexes of their Fabs with CD4-triggered gp120 antigen. CH54 Fab crystallized with gp120_93TH057_ core_e_+N/C and M48U1 in space group C2, a = 174.8 Å, b = 42.2 Å, c = 119.8 Å, and β = 115.2°. Crystals diffracted to 2.9 Å and contained 1 complex in the asymmetric unit. CH55 Fab crystallized as Fab alone, space group P1 with a = 74.0 Å, b = 74.0 Å, c = 75.9 Å, α = 106.8°, β = 108.4°, and γ = 91.8°, and in complex with gp120_93TH057_ core_e_ in space group P6_1_22 with a = 90.6 Å, b = 90.6 Å, c = 409.0 Å, and γ = 120.0°. Crystals diffracted to 2.46 Å and 3.8 Å with 3 Fabs or 1 complex in the asymmetric unit, respectively. Data collection and refinement statistics are detailed in Table 1.

**FIG 3:**
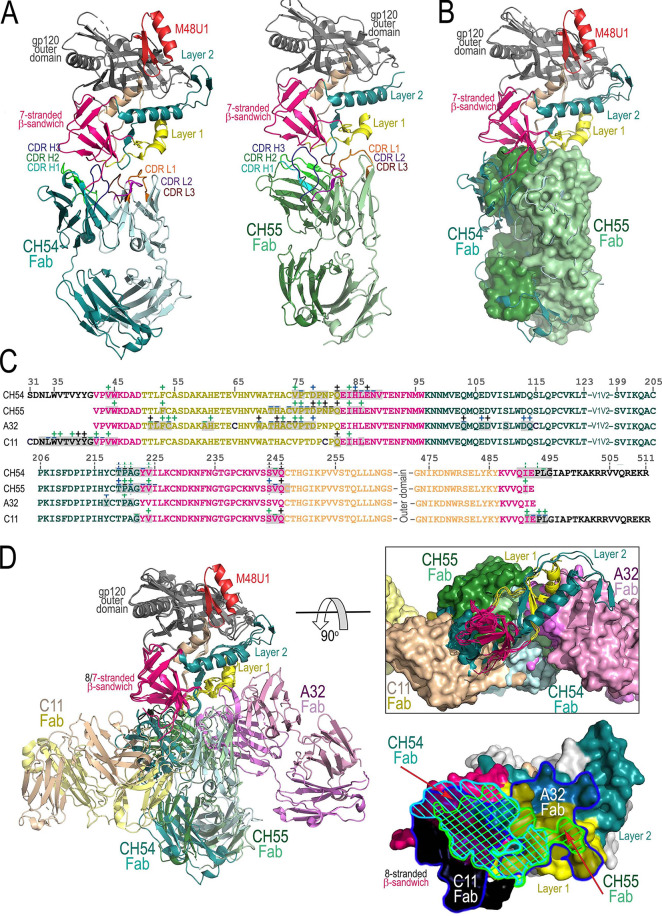
Crystal structure of CH54 Fab-gp120_93TH057_ core_e_+N/C-M48U1 and CH55 Fab-gp120_93TH057_ core_e_ complex. (A) The overall structures of the complexes are shown as ribbon diagrams. The gp120 inner domain elements and CDRs are colored as in Fig. 1. (B) Structural alignment of the complexes with CH54 and CH55 Fabs. Complex structures were aligned based upon gp120, and the molecular surface is displayed over the CH55 Fab. (C) Epitope footprints of CH54 and CH55 compared to the epitope footprints of A32 and C11. The gp120 residues involved in Fab binding (gp120 buried surface and contact residues) are shown over the primary sequence of gp120. Contributing residues are defined and labeled as in Fig. 1C. (D) Co-localization of CH54 and CH55 binding sites relative to the A32 and C11 recognition sites. The structures of complexes of gp120 antigen with the Fab of CH54 and CH55 were superimposed based upon gp120 and onto the gp120 antigen complexes of C11 Fab and A32 Fab (PDB code 4YC2). A blowup view with a 90° rotation of the superimposition is shown with molecular surfaces displayed over the Fabs. CH54 and CH55 epitope footprints are plotted on the gp120 surface (bottom right: CH54 footprint, blue lines; CH55, cyan lines) with layers colored as in panels A and B with A32 and C11 epitope footprints shown in blue.

Both CH54 and CH55 bind gp120 similarly, combining elements of the 7-stranded β-sandwich to make their epitope with additional contacts to gp120 inner domain mobile layers 1 and 2 (Fig. 3; see also Fig. S4 and Table S2). However, whereas CH54 depends more on contact to the β-sandwich (42% of the total gp120 BSA, residues 44, 45, 83 to 89, 223, 224, 244 to 246, 491, and 492) than CH55 (27%, residues 44, 45, 84 to 86, 223, 224, 244 to 247, and 491), and CH55 involves more residues of layer 1 (58%, residues 53, 71,72, and 74 to 82) than CH54 (26%, residues 53, 75, 76, 78 to 80, and 82), both also contact layer 2, with CH54 deriving 12% of its total gp120 BSA from layer 2 (residues 219 to 222) and CH55 deriving 15% (residues 219 to 222). Intriguingly, although CH54 was crystallized in complex with gp120 core_e_ with full-length N and C termini, the N terminus was completely disordered and not visible in the structure. In contrast, a relatively large part of the C terminus (residues 493 to 495) was ordered and contributed to CH54 gp120 BSA (approximately 20%). In both complexes, most of the contacts to the β-sandwich are made by the heavy chain of the Fab, with all three heavy-chain CDRs of CH54 contributing to binding and the CDR H3 of CH55 providing the majority of its contacts (Fig. 3A and S4 and Table S2). Interestingly, we found much of the CDR H3 disordered in the unbound CH55 Fab structure (Fig. S2) even given its modest length of 13 amino acids, implying that its structure rearranges upon binding to gp120. The vast majority of contact to layers 1 and 2 are mediated by the light chain for both CH54 and CH55, with CDR L1 and L3 contributing roughly equally for both antibodies (Fig. 3B; and S4 and Table S2). In total, CH54 buries 2,121 Å^2^ (1,095 Å^2^ from gp120 and 1,026 Å^2^ from Fab) and CH55 buries 1,906 Å^2^ (984 Å^2^ from gp120 and 922 Å^2^ from Fab).

10.1128/mBio.00208-20.4 FIG S4 CH54- and CH55-gp120_93TH057_ core_e_ antigen interface. The molecular surface is displayed over gp120 molecule and colored grey for gp120 outer domain, pink for 8-stranded β-sandwich, yellow for layer 1, and cyan for Layer 2. The N terminus of gp120 (residues 33 to 42) is colored in black. CH54 Fab (left) and CH55 Fab (right) residues that contribute to gp120 binding are shown as sticks. Complexes are shown in the same orientation. Download FIG S4, PDF file, 0.1 MB. Copyright © 2020 Tolbert et al. 2020 Tolbert et al. This content is distributed under the terms of the Creative Commons Attribution 4.0 International license.

As already mentioned, the epitope specificities of CH54 and CH55 were classified as cluster A and A32-like based on the ability to compete A32 binding to the AE.A244 gp120 envelope glycoprotein and the ability of A32 to block ADCC by these antibodies (25). Cross-competition with the second prototype antibody of cluster A region, mAb C11, was not tested. To confirm this initial epitope assessment and check if any elements of the C11 epitope region could be involved in forming the CH54 and CH55 antigen contact site, we analyzed antigen complex structures of CH54 and CH55 Fab in the context of Env antigen complexes formed by both prototype cluster A mAbs: A32 and C11 (Fig. 3C and D). As shown in Fig. 3D, superimposition of the complexes (based on gp120 molecule), mAb CH54 and CH55 bind to the gp120 region that overlaps the binding sites of both A32 and C11. First, CH54 and CH55 utilize many but not all of the same gp120 contact residues (residues 53, 75, 76, 78, 9, and 219 to 221 for CH54 and residues 53, 71 to 78, and 219 to 221 for CH55) of layer 1 and 2 that are used by A32 in gp120 recognition (residues 51, 52, 54, 60, 61, 69, 102, 103, 106, 107, 110, 113, 114, and 217 being unique for A32 and 79 to 80, 82, and 222 or 79 to 82 and 222 being unique to CH54 or CH55, respectively) (Fig. 3C). Second, both CH54 and CH55 interact with the gp120 inner domain 7-stranded β-sandwich, competing with C11 for residues in this region (residues 83, 84, 86, 223, 224, 244 to 246, 491, and 492 for CH54 and residues 44, 83, 84, 86, 223, 224, 244 to 246, and 491 for CH55). Furthermore, when gp120 is used to superimpose their structures, CH54 and CH55 overlap, falling midway between C11 and A32 and combining elements of both epitope classes (Fig. 3D, right). Interestingly, CH54 relies more on binding to the inner domain β-sandwich and gp120 C terminus and, in this regard, is more similar to C11 than CH55. However, neither CH54 nor CH55 interact with the gp120 N terminus, which in the antigen complex structure of C11 docks to the 7-stranded β-sandwich to form the 8th strand of the sandwich (Fig. 1).

**FIG 4:**
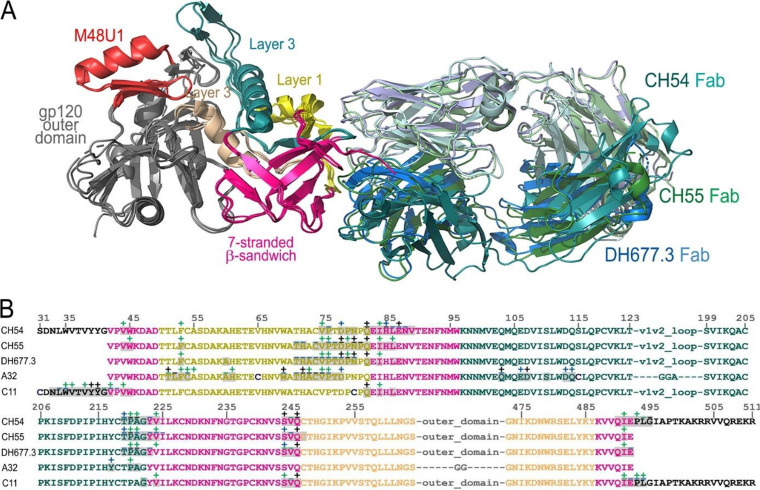
Structural comparison of Env antigen complexes of CH54, CH55, and DH677.3. (A) Structures of CH54 Fab-gp120_93TH057_ core_e_+N/C-M48U1, CH55 Fab-gp120_93TH057_ core_e_, and DH677.3 Fab-gp120_93TH057_ core_e_+M48U1 (PDB code 6MFP) are superimposed based upon gp120 core_e_ and shown as ribbons. (B) Epitope footprints of CH54, CH55, and DH677.3 compared to the epitope footprints of A32 and C11. The gp120 residues involved in Fab binding (gp120 buried surface and contact residues) are shown over the primary sequence of gp120.

Structural alignments of CH54, CH55, and DH677.3 antigen complex structures (Fig. 4) indicate that DH677.3 bears great resemblance to the mode of antigen recognition of both CH54 and CH55. Epitope footprints and structural alignment analyses confirmed a large degree of overlap between the CH54, CH55, and DH677.3 epitopes that includes involvement of the same gp120 contacts to the inner domain β-sandwich and mobile layers 1 and 2, with a similar Fab angle of approach to the antigen (Fig. 4B). DH677.3, like CH54 and CH55, contacts the gp120 inner domain 7-stranded β-sandwich with no contacts to the gp120 N terminus.

### Cluster A antibodies induced by RV144 vaccine and natural infection differ in recognition of the gp120 N terminus.

**FIG 5:**
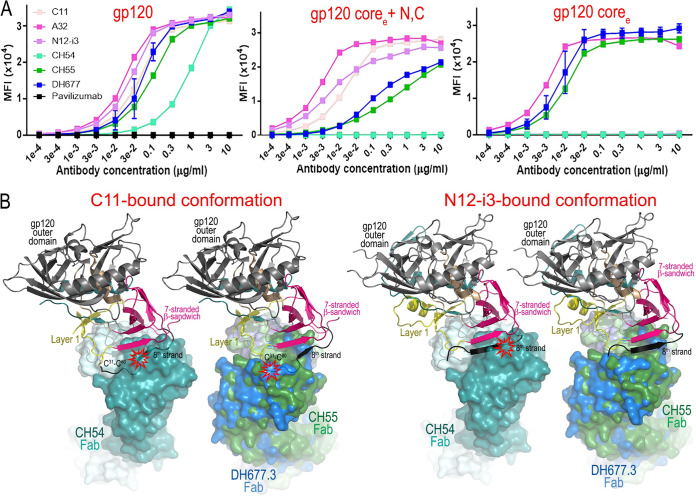
Dependence of cluster A antibodies upon gp120 N terminus recognition. (A) Binding of CH54, CH55, DH677.3, A32, and C11 to GFP-CEM-NKR-CCR5 SNAP cells coated with gp120 variants: gp120, full-length monomeric gp120; gp120 core_e_, gp120 construct with V1V2 and V3 loops and N and C termini removed; gp120 core_e_+N/C, gp120 core with incorporated N and C termini stabilized in C11 conformation by C-31 to C-80 disulfide bond. (B) Structural alignments of CH54, CH55, and DH677.3 onto C11-and N12-i3 antigen-bound gp120 conformations. CH54 Fab-gp120_93TH057_ core_e_+N/C-M48U1, CH55 Fab-gp120_93TH057_ core_e_, and DH677.3 Fab-gp120_93TH057_ core_e_ complexes were aligned based upon gp120 and placed onto the C11 Fab-gp120_93TH057_ (S31C, N80C) core_e_+N/C gp120 complex (left) or the N12-i3 Fab/N5-i5Fab-gp120_93TH057_ core_e_+N/C (PDB code 5W4L) (right). The molecular surface is displayed over CH54, CH55, or DH677.3 Fab molecules, and gp120 from the C11 or N12-i3 complexes are shown as ribbons. The possible steric clashes are indicated in red.

Structural data indicate that neither of the vaccine-induced antibodies CH54, CH55, nor DH677.3 involve the N terminus of gp120 for binding and therefore rely on the recognition of 7-stranded β-sandwich conformation of the gp120 inner domain. In this regard, they differ from C11 and C11-like antibody N12-i3, antibodies induced by natural infection, which recognized the 8-stranded β-sandwich (Fig. 2). In addition, CH54 and C11 also rely more on interactions with residues in the gp120 C terminus (Fig. 4). We showed previously that the 8-stranded β-sandwich is most likely formed at the late stages of HIV entry, as the gp120 N terminus must first be released from the interactions with gp41 promoter of the Env trimer (5). In contrast, the 7-stranded β-sandwich is fully formed in the Env trimer (33), and its exposure to the immune system is possible in the early stages of structural rearrangement induced by host receptor CD4 binding. Therefore, it can be speculated that during the process of CD4 engagement and the subsequent structural rearrangement of Env, the 7-stranded β-sandwich conformation exists for a longer time than the 8-stranded β-sandwich. To determine if the C and/or N termini of gp120 contribute to CH54, CH55, or DH677.3 binding and which gp120 conformations these antibodies preferentially recognize, we tested the antibodies’ ability to bind to target cells coated with different gp120 variants (Fig. 5A). In this assay, GFP-CEM-NKR-CCR5-SNAP cells were coated with monomeric full-length gp120, gp120 core_e_ (gp120 extended core as described in reference 2 with residues forming the N and C termini and residues from the V1V2 and V3 loops removed) and gp120 core_e_+N/C with S31C, N80C mutations (gp120 core_e_ with residues forming the N and C termini added and stabilized in the 8-stranded β-sandwich conformation by an added disulfide bond). In addition, antibody binding was also tested by SPR to the same gp120 variants immobilized on the sensor chip (Table S3). As expected, all antibodies effectively bound to the full-length gp120, which was presented in the CD4 bound conformation on gp120-coated cells. gp120 core_e_ was recognized by A32 and the vaccine-induced antibodies CH55 and DH677.3 but not C11, N12-i3, or CH54, pointing toward the possibility that N and C termini are required for the formation of C11, N12-i3, and CH54 epitopes. Interestingly, binding data to the gp120 variant stabilized to form 8-stranded β-sandwich (gp120 core_e_+N/C with S31C and N80C) (Fig. 5A), confirming dependence of C11 and N12-i3 on this conformation, as both effectively recognized this variant. Surprisingly, vaccine-induced CH55 and DH677.3 also recognized this variant, albeit with lower affinity, whereas CH54 did not. These data indicate that CH55 and DH677.3 were most likely generated primarily against the 7-stranded β-sandwich in the absence of the gp120 N terminus, but they can accommodate the N terminus in the 8-stranded β-sandwich, the conformation preferentially recognized by C11 and other C11-like antibodies. In contrast, CH54 targets the 7-stranded β-sandwich and, to some extent, the gp120 N and C termini, however, in a conformation that differs from that of the C11, 8-stranded β-sandwich fold. Interestingly, as shown in Fig. 5B, structural alignments of CH54, CH55, and DH677.3 antigen complexes on antigen-bound C11 (left) and N12-i3 (right) complexes reveal possible steric clashes between each vaccine-induced mAb (mediated through the heavy chain) to the 8th strand of the gp120 β-sandwich-stabilized C-31 to C-80 disulfide but, with the exception of CH54, not to the unconstrained N12-i3 conformation. The RV144 mAb CH55 and the RV305 mAb DH677.3 approach the gp120 antigen in a way which can accommodate the 8-stranded β-sandwich conformation, as seen in the N12-i3-gp120 antigen complex, with no steric clashes. The N12-i3-bound conformation of gp120 is not constrained by a C-31 to C-80 disulfide bond and therefore may be more representative of the native conformation of Env occurring *in vivo*.

10.1128/mBio.00208-20.7 TABLE S3 Binding kinetics of mAb C11, CH54, CH55, and DH677.3 to gp120 core_e_+N/C termini and gp120 core_e_+N/C termini with S31C/N80C mutation measured by SPR. The assay was run by passing Env glycoproteins over the immobilized antibody at 0 to 200 nM concentrations as described in Materials and Methods. The binding kinetics (association rates [*k_a_*], dissociation rates [*k_d_*], and affinity constants [*K_D_*]) were calculated with the BIAevaluation software. Standard deviations of *k_a_*, *k_d_*, and *K_D_* for two experiments are shown. While CH54, CH55, and DH677.3 can bind effectively to the gp120_93TH057_ core_e_ and the gp120_93TH057_ core_e_+N/C, their recognition of the C11 conformation mutant core_e_+N/C is diminished. The main effect of the mutation for C11 is to decrease the dissociation constant, but since the association constant for the mutant is also slower, the affinity is roughly equivalent between the two gp120s; in contrast, removal of the N terminus eliminates C11 gp120 binding (data not shown). CH54, CH55, and DH677.3 all have higher dissociation constants for the mutated gp120, which is partially explained by the fact that all three antibodies also use N80, one of the residues in the stabilizing disulfide, as an epitope contact residue. Since the association constant to the mutant gp120 is also higher, there is only a slight drop in affinity. Adding the N and C termini to wild-type gp120 decreases the association constant for CH54, CH55, and DH677.3. The slower association constants for the full-length gp120 are likely due to the N terminus conformation which can be disordered or in the C11 or N12-i3 conformations in the β-sandwich. CH55 and DH677.3 both have slower dissociation constants, which compensates for the slower association, but CH54 does not due to clashes with the N terminus. CH54 clashes outweigh any benefit from added contact residues at the C terminus, resulting in a lower affinity. CH55 and DH677.3 are less affected by the conformation of the N terminus. The high dissociation coupled with the low association and the added imposed geometric constraints to binding on a cell surface likely account for CH54’s poor binding and ADCC to gp120 coated cells. Download Table S3, DOCX file, 0.1 MB. Copyright © 2020 Tolbert et al. 2020 Tolbert et al. This content is distributed under the terms of the Creative Commons Attribution 4.0 International license.

### FRET-FCS-based epitope assigning of antibodies induced in RV144 trial.

**FIG 6:**
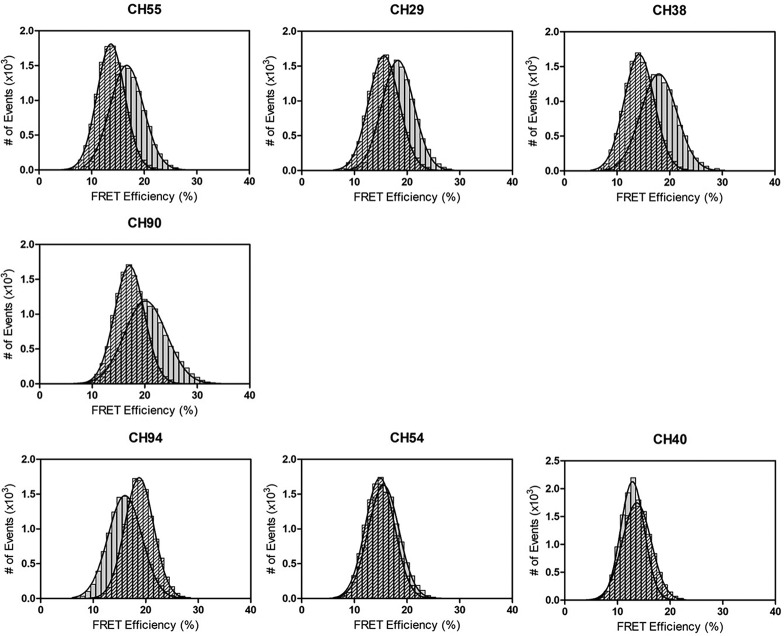
FRET-FCS definition of RV144 antibody epitopes. FRET histograms of donor (A488)-labeled C11 (gray) or A32 (shaded) Fabs against acceptor (A594)-labeled CH-series Fabs, and full-length single-chain gp120_BaL_-CD4 complex (FLSC) in solution as determined by the FRET-FCS approach (see Materials and Methods for details). From the FCS measurements, the diffusion coefﬁcient of dye-labeled Fab is ∼85 μm^2^/s. This diffusion has been signiﬁcantly decreased upon binding two Fabs to the FLSC. The diffusion coefﬁcient for this immune complex is ∼35 μm^2^/s.

To build upon our structural data and to further characterize the RV144 cluster A response, we mapped the epitopes of five other RV144 antibodies, CH94, CH29, CH38, CH90, and CH40 (25), with a FRET-FCS-based approach. In this method, two Fabs with different specificities compete in solution for binding to an antigen. Given the spatial requirements for FRET, a through-space dipole-dipole interaction occurs within the range of 20 to 90 Å between the donor-acceptor probes; FRET signals generated by paired fluorescently labeled Fabs can only occur via *cis* immunoreactivity with the same target antigen. We selected Fabs instead of mAbs to improve the precision of the measured donor-acceptor distance and to remain within the dynamic range of FRET efficiency measurements. The use of Fabs also facilitated comparisons to available structural data with Fab-antigen complexes. Previously, we demonstrated binding of donor-labeled C11-Fab and acceptor-labeled A32-Fab to a single full-length single-chain protein (FLSC) with a stoichiometry of C11/A32/FLSC of 1:1:1 by the FRET-FCS assay, with an average distance of 78 Å between C11 and A32 Fabs (3). Moreover, the binding of different cluster A Fabs to the FLSC was further confirmed by measurement of the diffusion coefficients of the immune complexes in solution. Consequently, the FRET-FCS approach with FLSC as an antigen was further developed to determine epitope specificity, defining A32-like, C11-like, or A32-C11 hybrid epitopes (3). Accordingly, we applied the FRET-FCS approach to define the epitope mapping of RV144 antibodies. Shown in Fig. 6 are the FRET histogram plots of A32- or C11-Fab pairs labeled with donor Alexa Fluor 488 (A488) or acceptor Alexa Fluor 594 (A594) for CH54, CH55, CH94, CH29, CH38, CH90, and CH40. Based on the FRET efficiency profiles for C11-A488 Fab with A594-labeled RV144 Fabs versus the A32-A488 Fab with A594-labeled RV144 Fabs bound to the FLSC, we can describe the epitope specificity of a given antibody. CH29, CH38, and CH90 showed FRET profiles most similar to that of CH55, with around 5% to 10% higher mean FRET efficiencies (equivalent to a change in distance of ∼4 to 10 Å for the A488-A594 donor-acceptor pair) for the C11-tested antibody pair than for the A32-tested antibody pair. Such profiles suggest that antibodies in this group overlap more with A32. In contrast, CH94 most likely recognizes the C11-like epitope, as determined by a higher mean FRET efficiency for the A32-CH94 pair than for the C11-CH94 pair. Finally, the FRET profile of CH40 is the most similar to the CH54 profile, with comparable mean FRET efficiencies to either C11 or A32. This suggests that CH54 and CH40 recognize similar epitopes and are more representative of the C11-A32 hybrid epitope specificity.

## DISCUSSION

Fc effector functions of antibodies such as antibody-dependent cellular cytotoxicity (ADCC) have been linked to protection from HIV-1 infection, with some of the strongest ADCC against HIV-1 entry targets mapping to the cluster A C1/C2 region of gp120 in Env. The cluster A region was first described (1) as the binding site for CD4-inducible (CD4i) antibodies isolated from natural infection that competed for binding to the CD4-triggered gp120 with mAb A32 or C11, two antibodies isolated from infected individuals in the early 90s (14, 15). These antibodies were capable of potent ADCC activity in an RFADCC assay of target cells sensitized with gp120 or AT2-inactivated virions (1, 29). Initial epitope assessment based on competition ELISA indicated that the A32 and C11 epitopes were not overlapping and were located within the gp41 interactive face of gp120. The CD4i mAbs described by Guan et al. (1) were later assigned as A32-like (if they competed for antigen binding only with A32), C11-like (if they competed for antigen binding only with C11), or as an A32/C11 hybrid (if they competed for antigen binding with both A32 and C11). To better understand the molecular details of these important ADCC targets, we determined the structures of A32 and several A32-like antibodies, described in reference 1 in complex with gp120 antigen (2–4). These studies confirmed that the A32 epitope region involves residues of gp120 inner domain mobile layers 1 and 2 in the CD4-bound conformation of gp120 in the occluded (within the native, untriggered HIV-1 Env trimer) face of gp120. The gp120 region that corresponds to the nascent A32 epitope is located in the center of the triggered HIV-1 Env trimer and is involved in the interactions with gp41. Here, to further the structural characterization of cluster A epitopes, we describe the epitope of C11, the second prototype antibody of the cluster A region isolated from natural infection. C11, similar to the previously characterized C11-like antibody N12-i3 (5), recognizes an epitope within the 8-stranded conformation of the gp120 inner domain β-sandwich. The 8th strand of the 8-stranded β-sandwich, which is emblematic of the C11 epitope, is formed by the gp120 N terminus that folds back, docking to the 7-stranded conformation of the β-sandwich. Similarly to epitopes in the A32 region, the C11 epitope is buried in the gp120-gp41 interface of the untriggered, native HIV-1 Env trimer. However, in contrast to the A32 region, which in its nascent form is involved in interactions with gp41, the C11 region can only be formed after gp120 disassociates from the trimer, because the N terminus is locked in a gp41 clasp within the trimer (33). The difference in location and conformation of the gp120 regions that form the nascent A32 and C11 epitopes suggest that these antibodies act at different stages of HIV Env trimer opening induced by CD4 engagement. The A32 epitope is most likely exposed before the C11 epitope, since it requires fewer Env trimer rearrangements that are very likely occurring in the initial steps of the CD4-mediated Env trimer opening. This opening, however, requires some motive force such as cell surface-bound CD4; soluble CD4 alone is not enough to expose these epitopes in the trimer (2–4, 36). On the other hand, the structural constraints of the C11 epitope suggest that it requires complete dissolution of the trimer, since adding the N terminus to the inner domain β-sandwich necessitates the release of the N terminus from the gp41 clasp (5).

In the RV144 vaccine trial, the only HIV-1 vaccine trial to show any efficacy against HIV-1 infection, the risk of infection was inversely correlated with ADCC in the presence of low Env-specific IgA titers. Antibodies induced by the vaccine mapped to the gp120 V2 loop and gp120 regions overlapping with the binding site of A32, an anti-cluster A antibody induced in natural infection (24, 25). V2 and A32-like antibodies synergized to provide levels of ADCC comparable to that observed in vaccinee plasma (27). Since the specificities of the RV144 antibodies were not characterized structurally, we performed a systematic analysis of their fine epitope specificities and compared them to those induced in natural infection. We also compared them to the anti-cluster A RV305 antibody DH677.3 (28). We were able to determine the crystal antigen complex structures of two RV144 antibodies, CH54 and CH55, and map the epitopes of five additional RV144 antibodies by FRET-FCS. CH54 and CH55, similar to DH677.3, bind the gp120 inner domain and include portions of the gp120 7-stranded β-sandwich and inner domain mobile layers 1 and 2 in their epitopes. While almost superimposable to one another in how they bind gp120, neither overlaps fully with either C11 or A32; their binding instead falls midway between the two, with elements of both the β-sandwich (C11 epitope) and layers 1 and 2 of the inner domain (A32 epitope). In antigen complexes determined by X-ray crystallography, neither of the vaccine-induced antibodies incorporate the gp120 N terminus in their epitope; in all cases, the epitope is formed by the 7-stranded conformation of the inner domain β-sandwich and not the 8-stranded conformation, which is emblematic of the C11 epitope. Interestingly, both the RV144 vaccine trial and the follow up RV305 vaccine trial used the same immunogens, which consisted of an ALVAC-HIV (vCP1521) prime with full-length gp120 and Gag, followed by an AIDSVAX B/E gp120 protein boost with an N-terminal 11-amino-acid deletion (del11gp120). The protein boost was administered in 4 doses over 24 weeks and was composed of gp120 protein lacking the gp120 N terminus that is essential for formation of the 8-stranded β-sandwich and the C11 epitope. The only immunogen that could elicit a C11-like epitope response, the full-length gp120, was only introduced in the ALVAC-HIV DNA prime, as the AIDSVAX B/E protein boost immunogen lacked the N-terminal 11 amino acids that form the 8th β-strand (37). This points toward the possibility that antibodies from both the RV144 and RV305 trials, although likely initially induced by the ALVAC-HIV (vCP1521) prime, matured through subsequent boosting with the truncated gp120 AIDSVAX protein variants. Although none of the vaccine-induced antibodies directly involve the gp120 N terminus to form their epitope in the antigen complex determined by X-ray crystallography, our modeling and binding analyses indicate that mAbs DH677.3 and CH55, but not CH54, are able to recognize and bind to the CD4-triggered gp120 core with the N terminus in its 8-stranded β-sandwich conformation. Binding data to gp120-coated cells show very similar binding patterns for CH55 and DH677.3, with strong binding to whole gp120 and gp120 core_e_ and decreased but still strong binding to core_e_ with added N and C termini stabilized in the C11 conformation by a C-31 to C-80 disulfide bond. Structural comparisons indicate that both CH55 and DH677.3, but not CH54, can accommodate the 8-stranded β-sandwich conformation, as seen in the N12-i3-gp120 antigen complex, with no steric clashes. This indicates that CH55 and DH677.3 primarily target the gp120 inner domain 7-stranded β-sandwich but can tolerate the 8-stranded β-sandwich conformation recognized by C11 and other C11-like antibodies. We have shown previously (5) that the 8-stranded β-sandwich conformation of the gp120 inner domain, emblematic of the C11 epitope, requires the release of the gp120 N terminus from the interactions with gp41 promoter in the Env trimer and therefore is most likely formed at the late stages of the CD4-induced structural rearrangement of the trimer that occurs on the target cell during virial entry or on freshly infected cells before CD4 is downregulated (33). In contrast, the 7-stranded β-sandwich is fully formed in the Env trimer, and its exposure to antibody recognition depends on structural rearrangements induced by the initial contacts of Env to the host receptor CD4.

Theoretically, this may give vaccine-induced antibodies a functional advantage over naturally induced antibodies in regard to “the window of opportunity” in which they can target new cell infections. Our results indicate that the RV144- and RV305-elicited mAbs CH55 and DH677.3 can bind both conformations, implying that they could function throughout the longer window-of-opportunity period in the entry process or on freshly infected cells before CD4 is downregulated. This could give them an advantage over 8-stranded β-sandwich conformation-dependent typical C11-like antibodies, which are limited to the later stages of the entry process. In contrast, the RV144 mAb CH54, which does not tolerate the 8-stranded β-sandwich conformation well, may bind less well in the later entry stages. Interestingly, the ability to bind different CD4i Env conformations appears to correlate with the potency and breadth of cluster A antibodies in their ability to evoke ADCC in two distinct systems: one designed to assay killing upon initial viral binding (gp120-coated RFADCC assay [29]) and the other during active viral infection (luciferase assay with cells infected with Nef-deficient IMCs [24, 27]). The luciferase reporter gene utilized in the IMC virus causes reduced Nef expression and, therefore, increased CD4 expression compared to natural viral-induced downregulation (38). However, it has previously been determined that Vpu is able to somewhat compensate for the lack of Nef, as CD4i antibodies are unable to bind to large portions of the envelope surface (39). In the gp120-coated RFADCC assay, the levels of ADCC induced were similar between all tested antibodies, with the exception of CH54 acting only at a higher antibody concentration. In contrast, much more diverse levels of ADCC were observed in the luciferase infected cell assay. In both clade-matched CEM235 and clade-mismatched BaL infected models, the RV305-induced antibody DH677.3 mediated the highest levels of lysis over the broadest range of concentrations. RV144-elicited antibodies CH54 and CH55 had poorer ADCC than both DH677.3 and antibodies induced during natural infection—A32 and C11. Differences between these two distinct assays are likely due to the fact that RFADCC presents only a single conformation of CD4-bound gp120, while infected cells present an array of antigen conformations, where cluster A epitopes can be fully or only partially accessible, depending on the nature of the CD4-triggering event mediated by CD4 remaining on the cell infected with Nef-deficient IMCs. Also, the geometry of how these CD4-triggered Env trimers are anchored to the infected cell surface may vary, adding more complexity to the cluster A epitope presentation on the infected cell surface. In agreement with our data, DH677.3 was also shown to mediate the most potent and broad ADCC activity among anti-cluster A antibodies isolated from RV305 vaccinees (28).

Taken together, these data point toward the possibility that the potency and breadth of cluster A antibodies targeting the β-sandwich region of the gp120 inner domain may be dictated by the ability to capture virions/HIV-infected cells in different entry stages/CD4-triggered Env conformations. Interestingly, of the vaccine-induced antibodies, DH677.3 showed the strongest binding to all three tested gp120 cores representing different conformations of Env. This correlated with the most potent ADCC in the luciferase infected cell assay in which the heterogeneity of the epitope conformation is higher. In contrast, binding analyses indicated that, differently from CH55 and DH677.3, CH54 is essentially unable to bind to gp120 core_e_ stabilized in the C11-bound conformation, and structural analyses indicate clashes with the gp120 N terminus of the 8-stranded β-sandwich seen in both the C11- and N12-i3-bound conformations of gp120. This may contribute to the lower ADCC potency of CH54, most clearly observed in the clade-matched CM235 infected model. Interestingly, three of the five RV144 antibodies we tested for epitope specificity using FRET-FCS showed profiles similar to those of CH55, indicating that they recognize overlapping epitopes. In contrast, only one antibody was shown to be similar to CH54 and only one showed full overlap with C11.

The exposure of cluster A epitopes on HIV-1 Env trimers is strictly CD4 dependent; they form and become available for antibody binding upon engagement of trimer in the complex with the cell surface CD4. Epitopes can also be exposed on infected cells by triggering the CD4 remaining at the cell surface post-infection or with a mixture of small CD4-mimetic compounds and coreceptor-specific antibodies (17, 21, 40, 41). Interestingly, existing evidence indicates that the A32 epitope is also only partially formed within the full-length monomeric gp120, as the secondary structural elements essential for its assembly such as the α1 helix are not formed/stable if gp120 is not complexed with soluble CD4 (4). C11 and CH55 both crystallized in the absence of the CD4-mimetic M48U1. In contrast, CH54 and DH677.3 were crystallized in its presence even though they largely overlap CH55 in binding to Env antigen. While in an intact Env trimer each would require CD4 to expose their epitopes, in the context of monomeric gp120, these epitope conformations do not appear to depend on CD4. CH54, CH55, and DH677.3 bind to the gp120 inner domain 7-stranded β-sandwich and regions immediately adjacent, implying that this structural feature is a major component of the vaccine immunogen. The canonical A32 epitope, which consists primarily of the gp120 inner domain mobile layers 1 and 2 in their CD4-bound conformation, may be less well represented in either the ALVAC-HIV (vCP1521) prime or the AIDSVAX protein boost because they both only contain monomeric untriggered (in the context of CD4 binding) gp120 as components. Eliciting a canonical A32-like antibody response by vaccination will therefore likely require a different immunogen.

Recently, the risk of altering the immunogen was highlighted with the halting of trial HTVN 702 due to slightly higher rates of infection among the vaccinated group that among those receiving placebo. This trial was undertaken in South Africa with the same prime-boost regimen as RV144, but with altered gp120 and canary-pox vector components to clade C and, additionally, utilized a different adjuvant. As the residues targeted by the antibodies tested throughout this study are known to be extremely well conserved, it is unlikely that altering the clade would prevent the induction of similar antibodies, but the change in clade may have affected their frequency due to differences in how they were exposed to the immune system. RV144 used an 11-amino-acid-deleted clade B/E gp120 protein boost; the N terminus was replaced with a herpes simplex virus (HSV) gD sequence (37). HVTN 702 and HVTN 100, the phase 1 trial proceeding HVTN 702, used a bivalent clade C full-length gp120 protein boost without the N-terminal deletion and HSV gD sequence (42). These differences, along with clade-specific conformational differences in gp120, may have had an effect on the type of antibodies induced. As we have previously published (43), adjuvant choice also makes a difference in the elicitation of ADCC-capable antibodies. Recently, Schifanella et al. showed adjuvant choice and concentration had a strong influence on outcome for ALVAC-HIV B/E-induced protection in macaques (44). The adjuvant used for RV144 was an aluminum hydroxide gel, while HTVN 702 used MF59, an oil-in-water emulsion. These adjuvants are known to act via very different mechanisms and may not have elicited the same type of antibodies as seen in RV144 and RV305. An analysis of the antibody responses from HVTN 097, a phase 1 trial using RV144 vaccine components in South Africa, and from HVTN 100 and HVTN 702 shows differences in the type and quality of ADCC responses in HVTN 100 and HVTN 702 compared to those in HVTN 097 and RV144 (42, 45). Whether these differences are due to the change in vaccine components, the change in adjuvant, or differences in the South African population is unknown.

In summary, the majority of gp120 residues contributing to A32 and C11 epitopes are extremely well conserved (46). In the intact Env trimer, they are buried beneath the highly variable and heavy glycosylated gp120 outer domain forming the gp41 interactive face of gp120. These epitopes only become exposed after CD4 attachment to the Env trimer, with A32 epitope exposure preceding C11 in the entry process (5). Because they are strictly CD4 dependent, they are occluded and not accessible for antibody recognition on productively infected cells that are CD4 negative (9, 13, 17). So, while the high degree of epitope conservation ensures that they are broadly cross-reactive against many HIV strains, their window of opportunity to impact the virus is short. The hybrid epitopes recognized by the RV144 CH54 and CH55 and the RV305 DH677.3 mAbs use many of the same conserved gp120 residues used by A32 and C11 and thus likely share their broad cross-reactivity to different HIV strains. This residue conservation is both structurally important in the stability of the trimer and functionally important in CD4/coreceptor-initiated cell fusion, suggesting that mutation of many of these residues comes at a high fitness cost (46, 47). DH677.3 was found to be particularly broad and potent against HIV, not only during viral entry but also against cells infected with Nef-deficient IMCs. The elicitation of antibodies like DH677.3 may therefore augment the efficacy of the RV144 vaccine trial. An additional protein boost with conformationally constrained gp120 locked in the C11-bound conformation would likely drive the antibody response toward a more DH677.3-like specificity, therefore increasing the range of gp120 conformations the antibody can bind and potentially increasing the effectiveness of the vaccine.

## MATERIALS AND METHODS

### Protein preparation and complex crystallization.

C11 and CH55 Fabs were crystalized at a concentration of ∼10 mg/ml. Clade A/E 93TH057 gp120 core_e_, or gp120 core_e_+N/C (gp120_93TH057_ residues 42 to 492 or 31 to 511, respectively [Hxb2 numbering]), lacking the V1, V2, and V3 variable loops and containing an H375S mutation to allow binding of the CD4-mimetic M48U1 (48) were used to obtain crystals of the Fab-antigen complexes. CH55 Fab was crystallized with gp120_93TH057_ core_e_ and CH54 with gp120_93TH057_ core_e_+N/C. C11 Fab was crystallized with a mutant version of gp120_93TH057_ core_e_+N/C that added a stabilizing disulfide bond between residues 31 and 80; S31C and N80C mutations were added with the QuikChange mutagenesis protocol (Stratagene). gp120_93TH057_ core_e_ proteins were prepared and purified as described in reference 2. Deglycosylated gp120_93TH057_ core_e_ proteins were first mixed with CD4-mimetic peptide M48U1 at a molar ratio of 1:1.5 and purified through gel filtration chromatography using a Superdex 200 16/60 column (GE Healthcare, Piscataway, NJ). After concentrating, the gp120_93TH057_ core_e_ or gp120 core_e_+N/C-M48U1 complexes were mixed with a 20% molar excess of Fab and passed again through the gel filtration column equilibrated with 5 mM Tris-HCl buffer (pH 7.2) and 100 mM ammonium acetate. The purified complexes were concentrated to ∼10 mg/ml for crystallization experiments.

### Crystallization.

Initial crystal screens were done in vapor diffusion hanging-drop trials using commercially available sparse matrix crystallization screens from Hampton Research (Index), Emerald BioSystems (Precipitant Wizard screen), and Molecular Dimensions (Proplex and Macrosol screens). The screens were incubated at 21°C and monitored periodically for protein crystals. Conditions that produced crystals were then further optimized to produce crystals suitable for data collection. C11 Fab was crystallized from 14% isopropanol, 140 mM sodium citrate, 7 mM HEPES (pH 7.5), and 30% glycerol, and CH55 Fab was crystallized from 25% polyethylene glycol 4000 (PEG 4000), 100 mM morpholineethanesulfonic acid (MES; pH 5.5), and 150 mM ammonium sulfate. The C11 Fab-gp120_93TH057_ (S31C, N80C)+N/C core_e_ complex was crystallized from 17% PEG 6000 and 100 mM sodium citrate (pH 5.0), the CH54 Fab-gp120_93TH057_+N/C core_e_-M48U1 complex was crystallized from 25% PEG 4000 and 100 mM MES (pH 5.5), and the CH55 Fab-gp120_93TH057_ core_e_ complex was crystallized from 15% PEG 6000 and 100 mM sodium citrate (pH 5.5). The C11 and CH55 gp120 complexes crystallized without M48U1 in the asymmetric unit despite having M48U1 during complex formation, suggesting crystal packing precluded M48U1 incorporation into the crystal. With the exception of C11 Fab crystals which were flash frozen directly in liquid nitrogen, crystals were briefly soaked in crystallization solution plus 18% to 20% 2-methyl-2,4-pentanediol (MPD) before being frozen in liquid nitrogen in preparation for data collection.

### Data collection and structure solution.

Diffraction data were collected at the Stanford Synchrotron Radiation Lightsource (SSRL) at beam line BL12-2 equipped with a Dectris Pilatus area detector. All data were processed and reduced with HKL2000 (49) or mosflm and scala from the CCP4 suite (50). Structures were solved by molecular replacement with Phaser (51). The C11 and CH55 Fab structures were solved based on the coordinates of the N12-i2 Fab (PDB 3QEG), and the complexes solved with refined Fab coordinates (C11 and CH55) or N12-i2 Fab in the case of CH54, gp120 (PDB 3TGT), and M48U1 (PDB 4JZW). Refinement was carried out with Refmac (52) and/or Phenix (53). Refinement was coupled with manual refitting and rebuilding with COOT (54). Data collection and refinement statistics are shown in Table 1.

### Structure validation and analysis.

The quality of the final refined models was monitored using the program MolProbity (55). Structural alignments were performed using the program lsqkab from the CCP4 suite (50). The PISA (56) webserver was used to determine contact surfaces and residues. All illustrations were prepared with the PyMol Molecular Graphic suite (http://pymol.org) (DeLano Scientific, San Carlos, CA, USA).

### Antibody binding.

Antibody binding was assayed against GFP-CEM-NKR-CCR5-SNAP target cells sensitized with three gp120 constructs: full-length gp120 (clade B, BaL strain), gp120 core_e_ (clade A/E, 93TH057 strain), and gp120 core_e_+N/C (clade A/E, 93TH057 strain). Target cells were incubated with 50 μg/ml gp120, washed and plated in V-bottom-well plates at 50,000 cells per well. Serially diluted antibodies ranging from 10 to 0.0001 μg/ml were then incubated with the cells for 20 min at room temperature before being washed twice. Cells were then incubated with A647-conjugated secondary antibody (diluted 1:1,000) for 20 min in the dark before washing and fixing in 1% paraformaldehyde. Samples were analyzed on an LSRII cytometer (BD Biosciences), and data analysis was performed using FlowJo vX.0.7 (Tree Star, Ashland, OR, USA).

### Antibody-dependent cellular cytotoxicity.

The ADCC activity of antibodies was assayed using the optimized rapid antibody-dependent cellular cytotoxicity (RFADCC) assay (29). GFP-CEM-NKR-CCR5-SNAP target cells were sensitized with recombinant HIV-1 BaL gp120 (50 μg/ml) to be used as target cells, and human PBMCs were used as effector cells. Serially diluted antibodies (ranging from 10 to 0.0003 μg/ml) were incubated with target and effector cells at a ratio of 50:1 effectors to targets. Following 2 h of incubation, the samples were washed and fixed with 1% paraformaldehyde. Events were collected on an LSRII cytometer (BD Biosciences) and analyzed using FlowJo software (Tree Star, Ashland, OR). All conditions were assayed in triplicates, and data are displayed as percent cytotoxicity, defined by the percentage of GFP-CEM-NKR-CCR5-SNAP target cells that lost GFP staining but retained the CCR5-SNAP tag surface staining.

### Infectious molecular clones.

The HIV-1 reporter viruses used were replication-competent IMCs designed to encode the *env* genes in *cis* within a Nef-deficient isogenic backbone that expresses the *Renilla* luciferase reporter gene (57). The subtype AE virus used was the NL-LucR.T2A-AE.CM235-ecto (IMC_CM235_; GenBank no. AF259954.1) (plasmid provided by Jerome Kim, US Military HIV Research Program) and was built on the 40061-LucR virus backbone; the subtype B BaL Env-IMC-LucR (IMC_BaL_, GenBank no. DQ318211) was built using the original NL-LucR.T2A-ENV.ecto backbone as originally described (58). Reporter virus stocks were generated by transfecting 293T cells with proviral IMC plasmid DNA, and virus titer was determined on TZM-bl cells for quality control (58).

### Infection of CEM-NKR-CCR5 cell line with HIV-1 IMCs.

CEM-NKR-CCR5 cells were infected with HIV-1 IMCs as previously described (59). A total of 1 × 10^6^ CEM-NKR-CCR5 cells (NIH AIDS Reagent Program, Division of AIDS, NIAID, NIH from A. Trkola [60]) were infected with a 50% tissue culture infective dose [TCID_50_]/cell of 48 for IMC_CM235_, 0.031 for IMC_BaL_, by incubation for 0.5 h at 37°C and 5% CO_2_ in the presence of DEAE-dextran (7.5 μg/ml). These amounts of virus were determined using our titration of the stock to achieve a frequency of p24^+^ (i.e., IMC-infected) cells greater than 10% and viability of target cells of >40%.

### Luciferase ADCC assay.

ADCC activity was determined by a luciferase (Luc)-based assay as previously described (24, 27). Briefly, CEM-NKR-CCR5 cells (NIH AIDS Reagent Program, Division of AIDS, NIAID, NIH from A. Trkola) (60) were used as targets after infection with the HIV-1 IMCs. PBMCs obtained from an HIV-seronegative donor with the heterozygous 158F/V and 131H/R genotypes for FcγR3A and FcγR2A (61, 62), respectively, were used as a source of effector cells and were used at an effector-to-target ratio of 30:1. All mAbs were produced using recombinant techniques and were generated using both a natural human IgG1 constant region and IgG1-containing alanine substitutions (S298A, E333A, and K334A) designed to enhance binding to Fcγ-receptor IIIa (FcγR3A) (63). Recombinant mAbs were tested across a range of concentrations using 5-fold serial dilutions starting at 50 μg/ml. Effector cells, target cells, and Ab dilutions were plated in opaque 96-well half-area plates and were incubated for 6 h at 37°C in 5% CO_2_. The final readout was the luminescence intensity (relative light units [RLU]) generated by the presence of residual intact target cells that have not been lysed by the effector population in the presence of ADCC-mediating mAb (ViviRen substrate; Promega, Madison, WI). The percentage of specific killing was calculated using the formula: percent specific killing = [(number of RLU of target and effector well − number of RLU of test well)/number of RLU of target and effector well] ×100. In this analysis, the RLU of the target plus effector wells represents spontaneous lysis in the absence of any source of Ab. The ADCC results are reported as area under the curve (AUC) and endpoint concentration. The AUC represents an integrated evaluation of maximum ADCC and lowest concentration (EC) required for detectable activity; it was calculated from dilution curves using a trapezoidal rule where specific killing below 15% was set to zero. The ADCC endpoint concentration (EC), defined as the lowest concentration of mAb capable of mediating ADCC in our *in vitro* assay, was calculated by interpolation of the mAb concentration that intersected the positive cutoff of 15% specific killing. The anti-Flu-hemagglutinin (HA) CH65 mAb (kindly provided by M. A. Moody) was used as a negative control.

### FRET-FCS-based epitope assessment.

Fabs of reference (A32 and C11) and tested (CH29, CH38, CH40, CH54, CH55, CH90, and CH94) antibodies were labeled with either donor (Alexa Fluor 488) or acceptor (Alexa Fluor 594) probes (Invitrogen mAb labeling kit). Dye-to-protein ratios were determined by measuring absorbance at 280 nm (protein) versus that at 488 or 580 nm (dye). The dye-to-protein ratios were between 1 and 2. This low level of dye labeling was used to minimally perturb the functionality of the protein. FRET measurements were performed with a confocal microscope (ISS Q2) equipped with a supercontinuum laser, acousto-optic tunable filter (AOTF), and laser-line cleanup filter in order to excite the molecules during their diffusion through the confocal volume. ISS VistaVision software was used to generate the FRET histogram and further analyses. FRET measurements were performed after forming immune complexes with a full-length single-chain protein (FLSC) with both donor-labeled Fab and acceptor labeled Fab. In all of these measurements, each Fab was used at a concentration of 1 μg/ml and FLSC at a concentration of 1.5 μg/ml. The immune complexes were made by incubating Fabs with FLSC at 20°C for 1 h. Fluorescence responses from the donor and acceptor molecules were separated by a dichroic beam splitter and detected by two avalanche photodiode detectors (APD) using time-correlated single photon counting and the time-tagged time-resolved (TTTR) mode of the Becker and Hickl SPC-150 module. High-quality bandpass (Chroma) filters were used for recording donor and acceptor fluorescence in two separate detection channels. The collected single photon data were binned by 10 ms in each channel (donor or acceptor), which resulted in intensity-time traces. Threshold values in each channel were used to identify the single molecule bursts from the corresponding background signal level. Fluorescence bursts were recorded simultaneously in donor and acceptor channels, and FRET efficiencies were calculated using *E* = *I_A_*/(*I_A_* + γ*I_D_*) where *I_D_* and *I_A_* are the sums of donor counts and acceptor counts for each burst, taking into account the possible difference in the detection efficiency (γ) in two separate channels (64). The donor-to-acceptor distance (*r*) in terms of efficiency of energy transfer (*E*) and Förster Distance (*R*_0_) is given by *r = R*_0_ [1/*E* – 1]^1/6^. We used the value of *R_0_* of 60 Å for the Alexa Fluor 488 (donor) and Alexa Fluor 594 (acceptor) pair for estimating the donor-to-acceptor distances. In addition to FRET measurements, we also performed autocorrelation analyses with FCS measurements to assess the *in vitro* binding of Fab fragments to FLSC. Consequently, we determined the translational diffusion coefficients of Alexa Fluor 488- or 594-labeled Fabs and the corresponding bound immune complexes from FCS measurements. It is important to note that the Fabs are not fluorescently labeled at a specific position. Hence, the distance determined by FRET measurements represents an average distance between the donor- and acceptor-labeled epitope probes.
