# Supplementary figures and images for: Recognition Patterns of the C1/C2 Epitopes Involved in Fc-Mediated Response in HIV-1 Natural Infection and the RV114 Vaccine Trial
Source: mBio. 2020 Jun 30;11(3):e00208-20. doi: 10.1128/mBio.00208-20 (PMC7327165; doi:10.1128/mBio.00208-20)

A

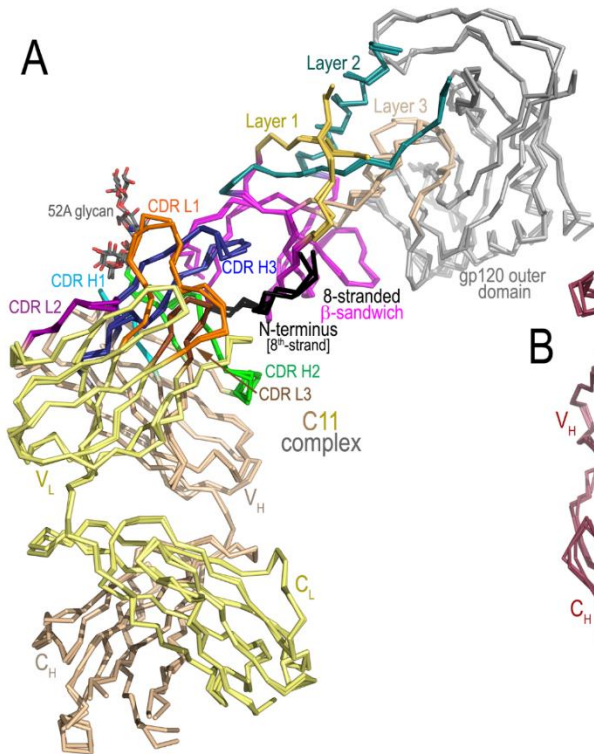

B

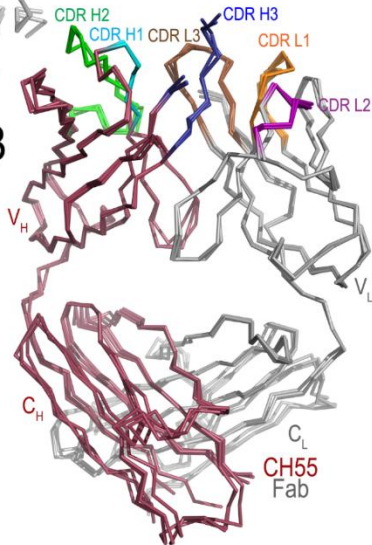

Supplement: FIG S1 [file mBio.00208-20-sf001.pdf]

A

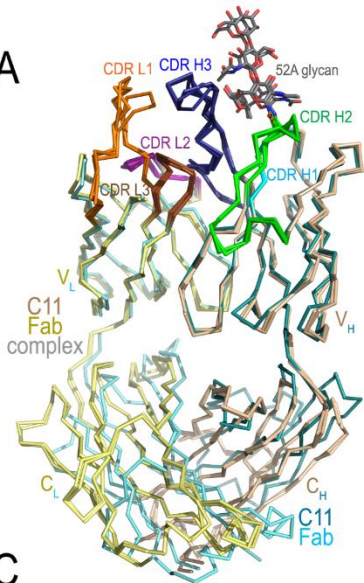

B

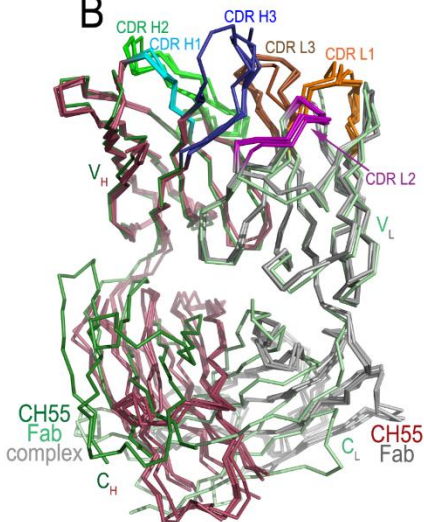

C

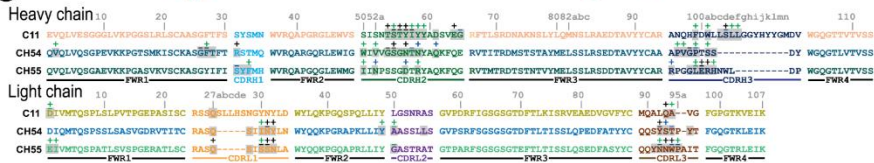

Supplement: FIG S2 [file mBio.00208-20-sf002.pdf]

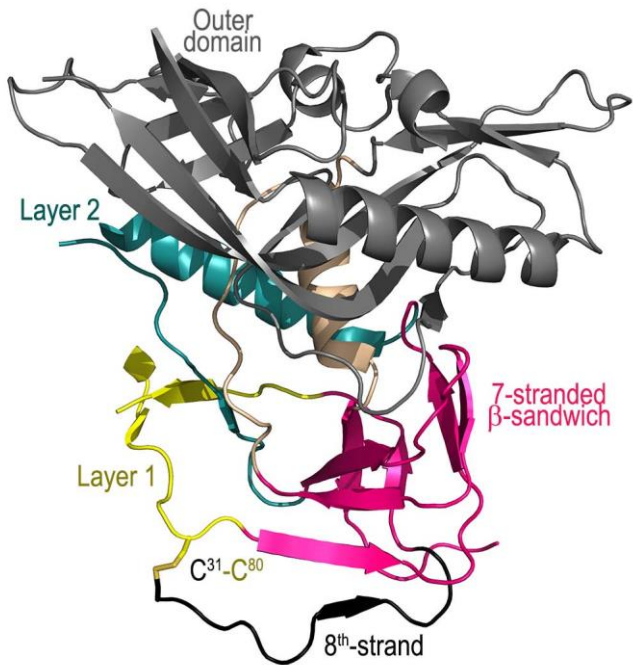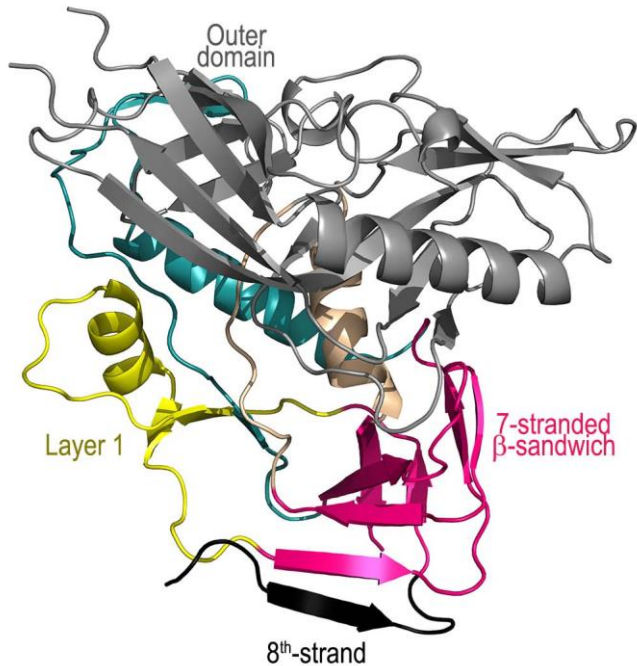

Supplement: FIG S3 [file mBio.00208-20-sf003.pdf]

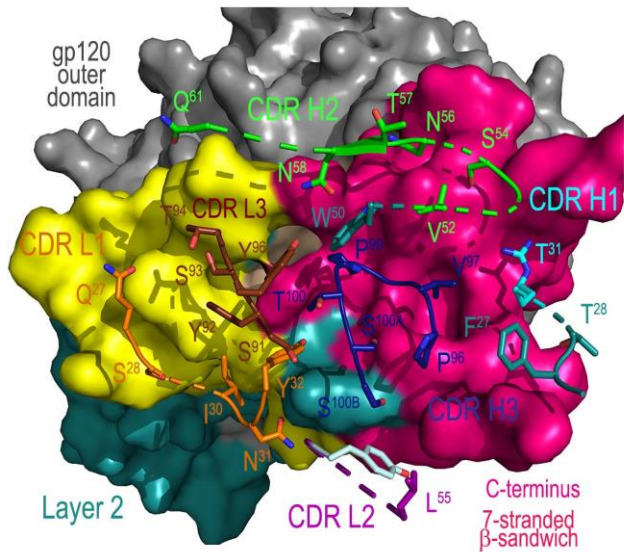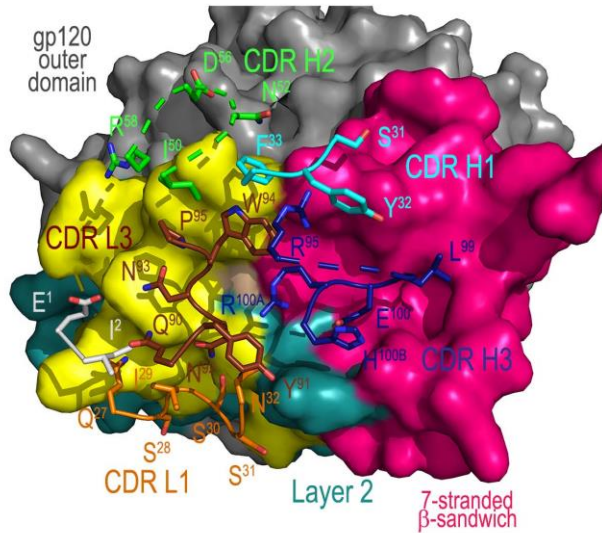

Supplement: FIG S4 [file mBio.00208-20-sf004.pdf]
