# Supplementary material for: Recognition Patterns of the C1/C2 Epitopes Involved in Fc-Mediated Response in HIV-1 Natural Infection and the RV114 Vaccine Trial
Source: mBio. 2020 Jun 30;11(3):e00208-20. doi: 10.1128/mBio.00208-20 (PMC7327165; doi:10.1128/mBio.00208-20)
Supplement: TABLE S1 [file mBio.00208-20-st001.docx]

|  | **gp120_93TH057_core_e_ +N/C** | **gp120_93TH057_(S31C, N80C) core_e_ +N/C** |
| --- | --- | --- |
| **mAb C11**  **K_D_ (M) x 10^-9^**  **k_a_(1/Ms) x 10^5^**  **k_d_(1/s) x10^-5^** | **0.15**  **145**  **166** | **0.25**  **17.7**  **41.2** |
