# Supplementary material for: Recognition Patterns of the C1/C2 Epitopes Involved in Fc-Mediated Response in HIV-1 Natural Infection and the RV114 Vaccine Trial
Source: mBio. 2020 Jun 30;11(3):e00208-20. doi: 10.1128/mBio.00208-20 (PMC7327165; doi:10.1128/mBio.00208-20)
Supplement: TABLE S2 [file mBio.00208-20-st002.docx]

|  | | **C11** Fab -gp120_93TH057 [S31C,N80C]_  core_e_+N/C | **CH54** Fab -gp120_93TH057_  core_e_+N/C-M48U1 | **CH55** Fab -gp120_93TH057_  core_e_ | **N12-i3** Fab- gp120_93TH057_ core_e_+N/C-M48U1  (5W4L) | **A32** Fab-ID2_93TH057_  (4YC2) |
| --- | --- | --- | --- | --- | --- | --- |
| **Buried Surface Area, Å²** | **gp120 total** | **768** | **1095** | **984** | **850** | **803** |
|  | gp120 N-terminus (31-42) | 406 | 0 | 0 | 0 | 524 |
|  | 7/8-stranded β-sheet | 598 | 466 | 267 | 0 | 754 |
|  | Layer 1 | 39 | 285 | 575 | 645 | 49 |
|  | Layer 2 | 20 | 136 | 143 | 205 | 0 |
|  | Layer 3 | 0 | 0 | 0 | 0 | 0 |
|  | **Heavy chain total** | **685** | **611** | **546** | **614** | **711** |
|  | FWR | 0 | 58 | 0 | 17 | 16 |
|  | CDR H1 | 0 | 99 | 75 | 103 | 39 |
|  | CDR H2 | 337 | 183 | 87 | 83 | 440 |
|  | CDR H3 | 348 | 271 | 384 | 411 | 216 |
|  | **Light chain total** | **126** | **415** | **376** | **234** | **142** |
|  | FWR | 46 | 37 | 38 | 0 | 0 |
|  | CDR L1 | 10 | 191 | 180 | 126 | 10 |
|  | CDR L2 | 0 | 33 | 15 | 0 | 0 |
|  | CDR L3 | 70 | 154 | 143 | 108 | 132 |
|  | **Heavy and light chain total** | **811** | **1026** | **922** | **848** | **853** |
