# Supplementary material for: Recognition Patterns of the C1/C2 Epitopes Involved in Fc-Mediated Response in HIV-1 Natural Infection and the RV114 Vaccine Trial
Source: mBio. 2020 Jun 30;11(3):e00208-20. doi: 10.1128/mBio.00208-20 (PMC7327165; doi:10.1128/mBio.00208-20)
Supplement: TABLE S3 [file mBio.00208-20-st003.docx]

|  | **gp120_93TH057_core_e_  – M48U1** | **gp120_93TH057_core_e_ +N/C – M48U1** | **gp120_93TH057_(S31C, N80C) core_e_ +N/C – M48U1** | **Fold change**  **(core_e_)** | **Fold change**  **(core_e_+N/C)** |
| --- | --- | --- | --- | --- | --- |
| **mAb C11**  **K_D_ (M) x 10^-9^**  **k_a_(1/Ms) x 10^5^**  **k_d_(1/s) x10^-5^** | **ND**  **ND**  **ND** | 0.15  145  166 | 0.25  17.7  41.2 | **–**  **–**  **–** | **1.7**  **8.2**  **4.0** |
| **mAb CH54**  **K_D_ (M) x 10^-9^**  **k_a_(1/Ms) x 10^5^**  **k_d_(1/s) x10^-5^** | 0.25  18.7  46.3 | 2.1  10.5  269 | 2.7  101  2640 | **10.8**  **5.4**  **57** | **1.3**  **9.6**  **9.8** |
| **mAb CH55**  **K_D_ (M) x 10^-9^**  **k_a_(1/Ms) x 10^5^**  **k_d_(1/s) x10^-5^** | 1.9  21.9  409 | 0.48  4.5  21.4 | 0.77  315  2460 | **-2.5**  **14.4**  **6.0** | **1.6**  **70**  **115** |
| **mAb DH677.3**  **K_D_ (M) x 10^-9^**  **k_a_(1/Ms) x 10^5^**  **k_d_(1/s) x10^-5^** | 0.19  21.7  40.7 | 0.42  7.8  32.7 | 1.0  52.5  516 | **5.3**  **2.4**  **12.7** | **2.4**  **6.7**  **15.8** |
